# Adjuvant Treatment for Surgically-Treated Cervical Cancer Patients: A Comprehensive Review

**DOI:** 10.3390/cancers17223710

**Published:** 2025-11-20

**Authors:** Stamatios Petousis, Aristarchos Almperis, Chrysoula Margioula-Siarkou, Frederic Guyon, Vasileios Pergialiotis, Nikolaos Thomakos, Konstantinos Dinas, Alexandros Rodolakis

**Affiliations:** 1 Gynaecologic Oncology Unit, 2nd Department of Obstetrics and Gynaeocology, Aristotle University of Thessaloniki, 54622 Thessaloniki, Greece; 2 Gynaecologic Oncology Unit, Institut Bergonié, 33000 Bordeaux, France; 3 Gynaecologic Oncology Unit, 1st Department of Obstetrics and Gynaeocology, National and Kapodistrian University of Athens, 11527 Athens, Greece

**Keywords:** cervical cancer, adjuvant treatment, guidelines, comprehensive review

## Abstract

Background/Objectives: Cervical cancer (CC) is the fourth most common gynecologic malignancy, disproportionately affecting women in low- and middle-income countries. Despite the effectiveness of HPV vaccination and screening strategies, CC poses a major global health issue, accounting for approximately 94% of annual deaths. Τhis review aims to summarize the current evidence regarding adjuvant treatment indications for surgically treated cervical cancer patients and identify areas where further research is required. Methods: After a literature search, a comprehensive review of the existing guidelines, clinical trials, and cohort studies related to cervical cancer treatment was conducted, focusing on the role of adjuvant therapy in patients classified as at low, intermediate, and high risk for recurrence, who may require no further treatment. Results: Adjuvant therapy is generally unnecessary for low-risk patients, while high-risk patients with lymph node invasion, parametrial involvement, or large tumor size require chemoradiation (CRT). Ιntermediate-risk patients fall into a grey zone, where the necessity of adjuvant therapy is still debatable. Guidelines emphasize the need for individualization in treatment strategies, since, based on the published studies, careful surgery alone and observation can provide similar outcomes to adjuvant therapy. Conclusions: This review emphasizes that achieving monotherapy remains pivotal to optimize outcomes and minimize overtreatment. Definitive adjuvant treatment is indicated for high-risk cases, and intermediate-risk patients may benefit from careful observation following adequate surgical intervention, pointing out the necessity of well-designed clinical trials.

## Simple Summary

Cervical cancer remains a concerning global health issue, where optimal therapeutic strategy is tailored to the stage of the disease. This review highlights the complexities of adjuvant treatment for surgically treated cervical cancer patients based on FIGO staging, advocating for individualized treatment to achieve monotherapy and minimize treatment-related morbidity. Early-stage and low-risk patients require no adjuvant therapy following adequate surgery. High-risk patients with lymph node metastasis, parametrial involvement, or positive margins need chemoradiotherapy. However, intermediate-risk patients remain a controversial issue. Low quality data fail to support the superiority of chemoradiotherapy over radiotherapy, while observation following adequate surgery seems an acceptable option. Enhanced preoperative diagnostics including MRI, diagnostic conization, and surgical staging may optimize risk stratification. The identified gaps in the current guidelines underscore the need for further research to optimize adjuvant treatment for various risk categories.

## 1. Introduction

Cervical cancer (CC) constitutes the fourth most frequent gynecologic malignancy. According to the WHO, it remains a significant health concern, with an estimated 660,000 new cases diagnosed in 2022 [1]. Despite the effective HPV vaccination, the level of screening, and early prevention strategies, cervical cancer still affects global health, especially in low- and middle-income countries, where it accounts for approximately 94% of the 350,000 annual deaths [1,2].

The therapeutic strategy for cervical cancer is predominantly tailored to the FIGO stage of the disease, based on clinical examination and preoperative imaging, while nodal status assessment, either surgically or with imaging, is crucial to discriminate locally advanced cases from early-stage disease that is eligible for operation [3,4]. Early diagnosis through screening expands the treatment options, enables fertility preservation, reduces the treatment intensity, and optimizes the quality of life. Early-stage disease treatment includes multiple therapeutic approaches such as radical surgery, fertility-sparing procedures (conization, trachelectomy), or primary radiotherapy, each with distinct trade-offs regarding fertility preservation, surgical morbidity, and radiation toxicity. A tumor size over 4 cm, clinically invaded parametria, and lymph node invasion, when diagnosed during the preoperative workup, comprise definitive criteria for non-operability, and the patient should be referred to definitive chemoradiation (CRT). However, there are marginal cases, especially those with a tumor size between 2 and 4 cm, in which the final histopathological criteria, such as lymphovascular invasion and the depth of stromal invasion may categorize patients as at increased risk for local or even distant recurrence. These cases pose significant therapeutic challenges, as avoidance of further adjuvant treatment may risk recurrence; meanwhile, the performance of adjuvant treatment may expose them to harmful radiotherapy (RT) side effects without profound benefit on their survival outcomes in certain cases [5,6,7]. Importantly, adjuvant treatment recommendations must be contextualized within regional healthcare infrastructures; disparities in HPV vaccination and screening programs, limited access to modern radiotherapy facilities, and resource constraints in low- and middle-income countries significantly influence the feasibility and external validity of evidence-based adjuvant strategies. The main objective of this comprehensive review is to summarize the current evidence about absolute indications and the optimal type of adjuvant treatment for surgically treated cervical patients, as well as to highlight grey zones in which further research is needed in order to achieve definitive conclusions. Specifically, this review provides a critical synthesis of the evolving intermediate-risk controversy, addresses practical approaches to achieve monotherapy through preoperative risk stratification, and offers clinical guidance in the complex landscape of post-operative cervical cancer management.

## 2. Materials and Methods

The aim of this comprehensive review is to gather and critically present the published evidence regarding the role of and necessity for adjuvant therapy in surgically treated cervical cancer patients, stratified according to low-, intermediate-, and high-risk categories for disease recurrence.

### 2.1. Search Strategy

This comprehensive review was conducted following the preferred reporting items for systematic reviews and meta-analysis (PRISMA) recommendations to critically evaluate the current literature and conduct a narrative and up-to-date review. MEDLINE, UpToDate, and PubMed electronic databases were searched up to May 2025 for relevant published articles discussing adjuvant therapy in patients with cervical cancer after primary surgical treatment. The search strategy was formed by combining the MeSH terms and keywords: “cervical cancer”, “adjuvant therapy”, “adjuvant treatment”, “chemoradiation”, “radiotherapy”, “low-risk”, “intermediate-risk”, “high-risk”, “recurrence risk”, “survival outcomes”, and “clinical guidelines”. These terms were combined using the Boolean operators “AND” and “OR” to create comprehensive search strings. Additionally, manual searching of the reference lists from identified articles and relevant review papers was performed to identify additional studies that may have been missed during the electronic database search.

### 2.2. Eligibility Criteria

Studies were considered eligible for inclusion if they reported on adjuvant therapy outcomes in cervical cancer patients stratified by recurrence risk categories (low-, intermediate-, or high-risk) following primary surgical treatment. Both prospective and retrospective studies, including randomized controlled trials (RCTs), cohort studies, case–control studies, and systematic reviews with or without meta-analyses, were considered eligible. Clinical practice guidelines from major oncology societies discussing adjuvant therapy recommendations for different risk categories were also included. References of the included studies were additionally cross-referenced to find additional publications eligible for inclusion in our review.

### 2.3. Exclusion Criteria

Studies were excluded if they lacked clear risk stratification of patients or did not provide specific outcomes data for different risk categories. Articles published in languages other than English, conference abstracts without full-text manuscripts, case reports, and expert opinions without original data were excluded. Studies involving patients with non-epithelial cervical malignancies, those with concurrent malignancies, or studies lacking clear definitions of risk categories were excluded from the analysis. Studies focusing on neoadjuvant therapy, palliative treatment, or recurrent disease management were excluded unless they provided relevant comparative data on adjuvant therapy approaches.

### 2.4. Study Selection Process and Results Organization

**Figure 1:**
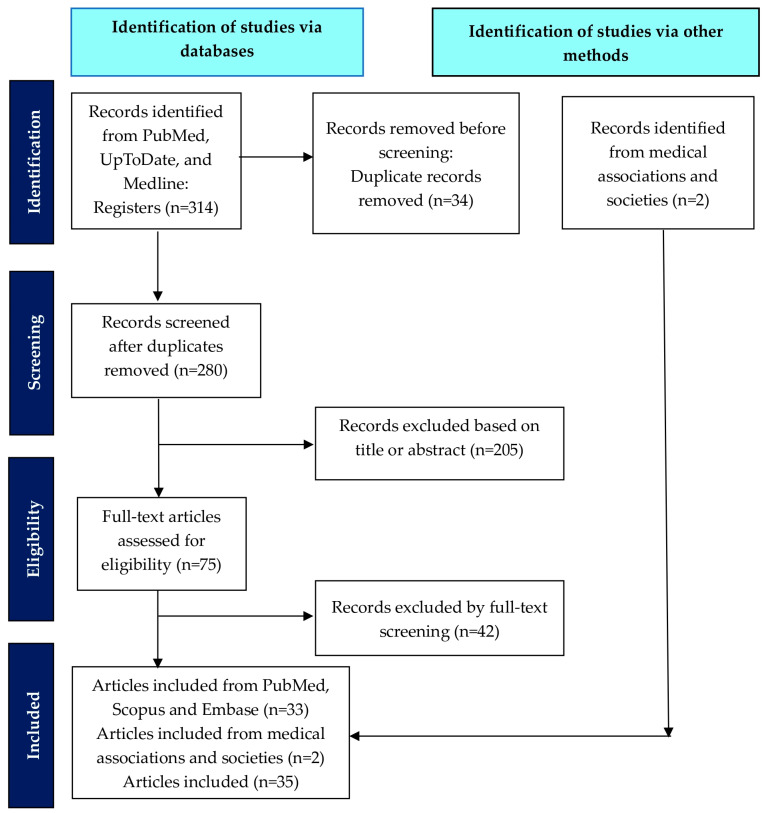
Flowchart of the study selection.

The retrieved articles were transported to reference management software (Zotero 6.0.30) for identification of duplicates and subsequent analysis. Two of the authors conducted a thorough assessment of each manuscript in full text, excluding the studies deemed non-applicable to the present review. The final evaluation of study inclusion was performed by 2 reviewers (S.P. and A.A.) according to the predefined eligibility criteria. The final included studies were organized into three main categories based on risk stratification: (a) low-risk patients, (b) intermediate-risk patients, and (c) high-risk patients. Within each risk category, studies were further subcategorized by study design: (1) randomized controlled trials, (2) prospective cohort studies, (3) retrospective cohort studies, and (4) systematic reviews and meta-analyses. This organizational structure facilitated comprehensive analysis of the evidence quality and consistency across different risk categories and study designs. The methodological quality of the included studies was assessed using design-specific tools: Cochrane Risk of Bias 2 for randomized controlled trials, Newcastle–Ottawa Scale for cohort studies, and AMSTAR 2 for systematic reviews and meta-analyses. Two independent reviewers (S.P. and A.A.) performed the assessments. The randomized controlled trials demonstrated a low-to-moderate risk of bias, though earlier trials had limitations related to historical surgical practices. Prospective cohort studies showed a moderate risk of bias due to the single-institution design and selection bias. Retrospective studies, post hoc analyses, and population-based studies exhibited a moderate-to-high risk of bias from inherent confounding and treatment heterogeneity. Systematic reviews and meta-analyses had a moderate-to-high risk of bias, primarily from the inclusion of heterogeneous retrospective data. The evidence synthesis prioritized high-quality randomized trials, while acknowledging the observational study limitations. Finally, 35 published studies and society guidelines/recommendations regarding adjuvant treatment were included in the present review. The flowchart of the study selection is presented in Figure 1.

## 3. Results

### 3.1. Stage IA1-IA2 Disease

Surgical treatment is the standard approach in this category of patients. Based on ESGO guidelines, regarding IA1 disease, conization is considered a definite treatment without lymph node dissection for negative lymphovascular space invasion (LVSI), while sentinel lymph node biopsy (SLNB) might be considered, although not necessarily, for positive LVSI, based on the preoperative diagnostic conization. Regarding IA2 disease, both conization and simple hysterectomy are considered as an adequate treatment. In cases with negative LVSI, the possibility of sentinel node biopsy may be discussed, while in the case of positive LVSI, both the ESGO and NCCN guidelines suggest SLNB [3,4]. Both guidelines state firmly that there is no need for adjuvant therapy in the IA1-IA2 stages, provided there is adequate lymph node staging. However, in the case where lymph node invasion is detected post-operatively, the disease is upstaged to IIIC; therefore, there is an absolute indication for complementary treatment, namely CRT, since these patients are considered at high-risk for local or distant recurrence.

### 3.2. Stage IΒ1-ΙIA1 Disease

The basic therapeutic treatment, according to the ESGO guidelines, should be monotherapy [3]. The main reason for monotherapy lies in the high morbidity rates observed in cases of combined treatment. Specifically, the increased rate of grade 3–4 adverse events for treatment combination was demonstrated in an RCT performed by Seldis et al. [5], in which the authors reported a 6% rate in combined-treatment cases vs. only 2.1% in monotherapy group. Relative rates were also reported by Peters [6] et al. and Kim et al. [8]. However, we have to report that other retrospective studies reported similar rates between the two arms [9]. In any case, as there is no clear evidence from prospective RCTs that treatment combination is not significantly different from monotherapy in terms of grade 3–4 events; hence, the treatment strategy should be properly individualized to avoid a combination of treatments, either to operate or to provide CRT.

Even if the proper individualization of cases based on preoperative imaging and conization may identify cases in which monotherapy may not be performed, there are certain cases where the post-operative question of additional therapy may reasonably be posed. The ESGO guidelines categorize cases in groups of high, intermediate, and low risk for recurrence, which represent different clinical entities, even if they belong to same stage. ESGO 2018 criteria categorize patients using a binary model based on the LVSI status, the depth of stromal invasion, and the tumor size, simplifying the specific combinations that the historical Seldis criteria required. However, this has been revised in the 2023 revised ESGO guidelines, in which patients characterized by any or a combination of the former parameters are classified as intermediate-risk. NCCN effectively uses the Sedlis criteria as a baseline framework while acknowledging histology-specific considerations. The evolution from strict to more flexible criteria has progressively expanded the population receiving adjuvant therapy, potentially overtreating patients who would have otherwise been observed. The risk stratification criteria across guidelines and mapping to Sedlis criteria can be seen in Table 1.

### 3.3. High-Risk Patients

There are three certain indications for which patients are considered as for high risk for recurrence and necessitate adjuvant treatment based on the 2023 guidelines: lymph node invasion, involvement of vaginal margins, and parametrial involvement. Based on the ESGO 2023 guidelines, these patients pose an absolute indication for CRT. This is mainly based on the findings of the RCT performed by Peters et al. [6]. In this study, patients with the former criteria were randomly assessed to either only RT or CRT. Patients in the second group had significantly improved 4-year progression-free survival (PFS) and overall survival (OS) (63% vs. 80% and 71% vs. 81% respectively), thereafter indicating the need for CRT in surgically treated cases with the former indications. Additionally, boost with vaginal cuff brachytherapy is recommended in patients with vaginal or parametrial disease, as vaginal cuff recurrences account for nearly 15% of all recurrences after EBRT alone [6]. Regarding the role of adjuvant chemotherapy (ACT), few studies have tried to address the exact benefit of adding ACT to CRT or even performing ACT instead of CRT. Kim et al. prospectively studied high-risk patients who received additional CT to concurrent CRT, indicating no additional survival benefit [10]. Similar conclusions were reached by a systematic review and meta-analysis of 15 trials and 4041 patients with locally advanced disease, where no significant improvement in Disease-Free Survival (DFS) and OS was indicated [11]. Furthermore, in a recent prospective non-inferiority study, ACT alone emerged as favorable treatment alternative for early-stage patients with risk factors, offering similar DFS and OS outcomes with a better toxicity profile and quality of life [12]; however, the evidence is limited, and further studies are needed.

In conclusion, nodal invasion, parametrial invasion, and positive margins represent absolute indication for post-operative CRT over RT, as the randomized evidence strongly supports. However, prospective studies provide scant evidence of the survival benefit from adding adjuvant chemotherapy after CRT, although adjuvant chemotherapy alone may be non-inferior to CRT with a better tolerability profile. Table 2 presents the primary results of landmark trials in high-risk cervical cancer patients.

### 3.4. Intermediate-Risk Patients

This category of patients probably represents the most conflictual one regarding the need for adjuvant treatment. Even if adjuvant treatment may seem to be of profound benefit for certain categories of patients, an observation strategy has gained increasing interest and scientific support, as opposed to the absolute indication for post-operative RT.

The ESGO guidelines suggest that adjuvant RT “should be considered” in patients with combination of risk factors, namely substantial LVSI, tumor size over 2 cm, and significant stromal invasion. However, they also claim that, in the case of an adequate type of radical hysterectomy performed, observation might be an acceptable alternative, especially in centers with related experience [3].

On the one hand, there is the well-known RCT of Sedlis et al. [5], in which patients with a combination of risk factors, as shown in Table 3, were stratified to either adjuvant RT or no adjuvant treatment. This was probably the most well-designed RCT that indicated that the risk of recurrence was significantly lower for the complementary radiation arm compared to the no-further-therapy arm (15.3% vs. 27.9%). Post-operative pelvic RT resulted in significantly higher recurrence-free rate at 2 years compared to no further treatment (88% vs. 79%, *p* = 0.008) [5]. The published meta-analysis of Zhang et al. [13] is also in favor of adjuvant treatment. In this study, the authors demonstrated that DFS and OS were significantly improved in patients receiving adjuvant treatment independently from optional concurrent chemotherapy. However, data included both retrospective studies and RCTs, therefore enrolling low-quality data with a high concern of bias.

On the other hand, the Sedlis criteria have encountered significant concern during the recent years. According to those opposed to Sedlis, this RCT reflects the results of a previous era, with a potentially lower degree of expertise in cervical surgery. Since 1999, evidence has been published that did not support the absolute need for additional treatment in well-operated related cases. Specifically, Lahousen et al. [14] performed a prospective RCT between 1989 and 1995 in which they observed no significant difference in DFS and OS between chemotherapy, RT, and observation for cases based on a combination of negative prognostic factors. These authors concluded that a well-performed radical hysterectomy might be the most significant determinant of survival parameters. Tozzi et al., in their prospective cohort study of 74 IB3 cases, in which adjuvant treatment was omitted for patients who received an adequate type of radical hysterectomy, concluded that DFS rate was 89.7% and OS rate 93.1%, with an overall complication rate of 23.5% and no grade 4–5 complications in a median of 68 months of follow-up [15]. Even in the Sedlis study, no significant differences in overall survival were observed between the two groups, while similar remarks were also made by Rotman et al. [16] in 2006, in a post hoc analysis of the GOG 92 study, in which no difference was detected regarding the OS and DFS rates.

There is relatively adequate evidence, but with no prospective RCTs, to support a modern strategy of observation-only strategy for intermediate-risk patients. A recently published sub-analysis of the SCCAN study by Cibula et al. assessed retrospectively the role of adjuvant therapy (including RT or CRT) compared to no adjuvant therapy with only observation [17]. The combination of CRT with radical surgery was not associated with a longer 5-year DFS (83.2% and 80.3%, PDFS = 0.365) or a longer OS (88.7% and 89.0%, POS = 0.281) compared to radical surgery alone. Furthermore, Van der Velden et al. [18] retrospectively analyzed 161 patients who underwent only type C2 radical hysterectomy and demonstrated a 2.5% mortality from isolated loco-regional recurrence, with the 5-year Recurrence Free Survival (RFS) being 86.6% and the 5-year OS 90%, respectively. Other retrospective studies comparing observation only with adjuvant RT or CRT were similarly not able to demonstrate any survival benefit in this group of patients, although the results should be interpreted with caution because of possible differences in the classification system, the lack of accurate implementation of Sedlis’ criteria, and the heterogeneity in the treatment approach [19,20]. In addition, Gómez-Hidalgo et al. recently tried to evaluate the impact of adjuvant therapy compared to surgery only in terms of recurrence and mortality. Their systematic review and meta-analysis found no significant difference in the relative risk of recurrence (RR 1.49; 95% CI 0.81–2.75) and mortality (RR 1.34; 95% CI 0.71–2.54) between the two categories of patients [21]. Finally, Rogers et al. [22] also concluded that post-operative RT and CRT are not associated with a significant increase in OS, although women receiving radiation had an almost 40% decrease in disease progression at 5 years.

Regarding the role of concomitant chemotherapy, Kim et al. showed that CRT offered no additional survival benefit compared to RT alone in intermediate-risk patients who underwent radical hysterectomy with pelvic lymphadenectomy demonstrating similar 5-year RFS rates (90.8% vs. 88.9%, *p* = 0.63) and 5-year OS rates (95.9% vs. 91.0%, *p* = 0.29) [8]. Conflicting with the previously published data were the results of the recent systematic review and meta-analysis of 13 studies by Guo et al. [23]. Their data highlighted that adjuvant CRT offered better RFS in patients with multiple intermediate-risk factors compared to RT alone after radical surgery (odds ratio (OR) 3.11; 95% CI 1.04 to 4.99; *p* < 0.0001; i2 = 6%). However, a similar benefit was observed between both regimens in the presence of a single intermediate-risk factor (OR 1.80; 95% CI 0.96 to 3.36; *p* = 0.07; i2 = 0%). Further, the quality of evidence was low due to the inclusion of multiple retrospective studies.

In conclusion, it is still debatable whether post-operative RT has a significant impact on PFS in intermediate-risk patients, while no published relative study demonstrates a clear OS benefit. There is also low-quality evidence supporting the supremacy of CRT over RT for this category of patients, without related suggestions in the main guidelines [3].

It is mandatory also to highlight the need to balance the potential survival benefits against treatment-related morbidity when deciding on adjuvant therapy. Μodern intensity-modulated radiotherapy (IMRT) has significantly reduced acute toxicity compared to conventional techniques. When combined with brachytherapy boost, it allows targeted tumor dose escalation, improving local control while limiting toxicity. Brachytherapy boost is especially indicated in patients with residual or bulky tumors after external beam radiation, enhancing the treatment efficacy without significantly increasing the side effects. Τhis strategy balances the safety of oncologic outcomes with improved treatment-related morbidity profiles, especially in intermediate-risk cases [24,25].

In this context, the appropriate individualization of patients might be the best solution until definitive evidence is derived for that category of patients, in a continuously changing landscape. A concise summary of adjuvant therapy studies in intermediate-risk cervical cancer patients is presented in Table 4.

### 3.5. Low-Risk Patients

Low-risk patients are those characterized by a tumor size less than 2 cm, without LVSI, and a depth of invasion lower than 1/3. The ESGO guidelines do not pose any indication for adjuvant treatment in an adequately operated CC patient. Scharl et al. [26], in a large population-based study of 442 patients, observed that low-risk patients receiving adjuvant treatment presented comparable OS, but also significantly deteriorated PFS, compared to those with no adjuvant treatment. Similar conclusions were published by Sun et al., in a retrospective series of 208 patients, in which adjuvant RT resulted in a comparable survival rate (97.0% vs. 95.0%) or recurrence rate (4.0% vs. 4.7%) compared to patients without complementary treatment [27]. The authors concluded that their study reaffirmed evidence against complementary RTCT in low-risk early-stage cases.

In conclusion, it seems rather definitive that no adjuvant treatment is required in this category of patients under the condition of an adequately performed surgery. However, it is still debatable as to what should be defined as “adequate surgery” for this category of patients, also taking into consideration the recently published results of the SHAPE study [28]. Even in that case, it seems that there is no actual indication for adjuvant treatment under the condition of the absence of negative prognostic factors.

### 3.6. Cervical Cancer as an Incidental Finding Following Inadvertent Hysterectomy

The incidence of cervical cancer following a hysterectomy indicated for benign conditions remains unknown; its prevalence is considered to be extremely scarce [29]. Although, to date, data from large cohorts seem to favor hysterectomy as an alternative to radical hysterectomy for millimetric disease, namely stages IA1 and IA2, [30], it must be remembered that in contrast to a simple hysterectomy, the type A hysterectomy that is mainly indicated for these stages involves removal of the paracervix close to the cervix, without the need to deflect the ureter but leading to a surgical margin that maintains a minimum amount of the paracervical tissue, therefore, ensuring complete removal of the cervical bed [31]. The performance of this procedure is rare for most surgeons who are not routinely involved with gynecologic oncology procedures, thus calling into question the adequacy of the procedure even in the earlier stages of the disease.

Several issues arise when dealing with patients who had an inadvertent hysterectomy for otherwise benign conditions and present with invasive lesions in the final histologic analysis. First, one has to consider that post-operative RT may be associated with significant adverse effects that may involve up to one-third of these cases, with approximately 25% being related to severe complications [29]. Moreover, although the actual importance of the interval between primary surgery and adjuvant RT has been rarely evaluated [32], rapid access to adjuvant treatment must be considered essential in these cases.

Concerning the preoperative assessment of patients, at least one study denoted that the extent of suboptimal preoperative workup in these cases may vary considerably, as even cases that were handled for carcinoma in situ of the uterine cervix had locally advanced cancer [33], therefore, denoting the necessity for referral to specialized gynecologic oncologists. A multicentric study that investigated factors that were associated with the survival of patients who had a diagnosis of occult invasive cervical cancer after simple hysterectomy, indicated that a large proportion of patients were referred for the procedure following a conization that indicated positive resection margins in the presence of preinvasive disease or microinvasive cervical cancer (41.6%) [34]. The predictors of survival included the tumor width (using a cut-off value of 20 mm) and presence of superficial stromal invasion.

Therefore, upon diagnosis, further management steps should include referral to a gynecologic oncology center for addressing the issue with a multidisciplinary board after the histopathological review. A staging work-up (pelvic MRI ± PET-CT) evaluating the tumor width > 20 mm, deep stromal invasion, LVSI, and margins should follow [3]. Considerations like intermediate- or high-risk factors, the stage of disease, and the medical conditions of the patient, should be taken into account, to avoid combining further surgery and RT, due to highest morbidity. For T1a1 (any LVSI) and T1a2, LVSI-negative disease with clear margins, no additional treatment is required. T1a2 LVSI-positive or T1b1 with clear margins cases, require surgical lymph node assessment. For ≥T1b2 disease or positive margins, (chemo)radiotherapy is the treatment of choice (Grade B). Adjuvant treatment must be initiated within 4–6 weeks [32,34]. Patients should be counseled on the potential toxicities (≈30% overall, 25% severe) [33], while emphasizing that contemporary image-guided IMRT with strict organ-at-risk dose constraints can reduce the severe toxicity rates by 10–20% compared to historical approaches.

### 3.7. Potential Therapeutic Strategy Towards Achieving Monotherapy

**Figure 2:**
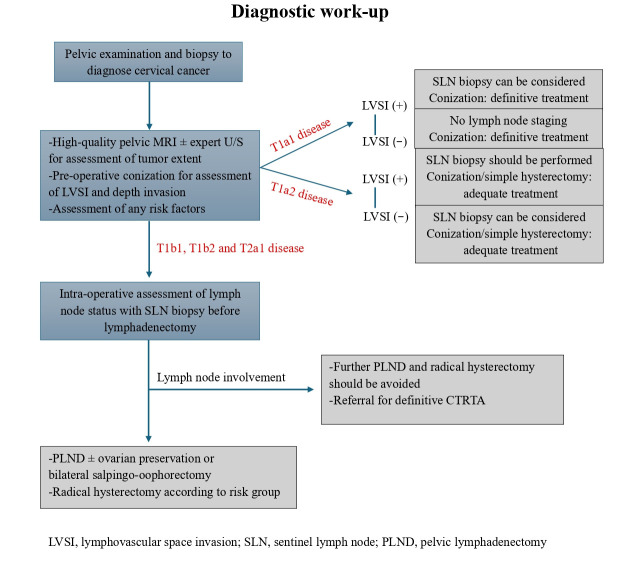
Diagnostic work-up towards monotherapy.

International guidelines still claim the absolute need to achieve monotherapy in the treatment of cervical cancer [3]. However, based on evidence, this does not seem to be followed or achieved as a strategy in the majority of cases. It is characteristic that Pan et al., in a series of 675 patients, reported that 51% of IB2 cases necessitated adjuvant treatment [35]. The main indications for adjuvant treatment were lymph node involvement in 17% and parametrial involvement in 9%. If we accept that monotherapy is indeed the primary goal of treatment, there should be strong efforts to significantly reduce the rates of adjuvant treatment in surgically treated cases. Potentially, the maximum a priori knowledge of histopathological details that may post-operatively pose the indication of adjuvant treatment could help to avoid combined treatment. In this context, diagnostic conization, suggested by the ESGO guidelines as essential diagnostic step before definitive treatment [3], may provide all the histopathological information about the LVSI and depth of stromal invasion, while the combination of MRI with ultrasound may give a precise estimation of the tumor size. Additionally, potential surgical staging in a previous surgical step to radical hysterectomy would permit the avoidance of need to perform combined treatment in the 17% of cases with finally invaded lymph nodes, while it might help to avoid the 10% of false frozen section, as mentioned in the Tozzi et al. study [16]. To summarize, initial management with diagnostic conization along with staging laparoscopic lymphadenectomy would be a potential strategy to reduce the rates of combined treatment. However, this strategy should be validated by large prospective studies before being implemented as the standard of care in daily practice, taking into consideration the difficulties it may pose on the level of the optimal use of the available surgical time and the consequent difficulty to perform a radical hysterectomy in a previously operated pelvic surgical field. Figure 2 depicts a potential clinical algorithm in order to achieve monotherapy in clinical practice.

## 4. Considerations for Adjuvant Therapy and Future Perspectives

A recent ESGO survey regarding the management of CC indicated that there is significant variability in the use, selection, and adjuvant treatment options across different centers and regions. The majority of participants (81%) considered a combination of tumor size, LVSI, and stromal invasion as indicators for adjuvant RT. Integrating additional prognostic factors, such as high-grade disease, non-squamous histology, age < 50 years, close margins, tumor size ≥ 4 cm, and suboptimal surgery or node dissection, were among the main recommendations. The consensus among respondents was the urgent need to incorporate additional variables alongside classic prognostic factors to refine the treatment decisions [36].

These concerns are reasonable, given the fact that parameters such as the histologic type are not taken into consideration for analysis. Levinson et al. [37] conducted an ancillary analysis of GOG studies 49, 92, and 141, focusing on stage I patients who underwent surgery without neoadjuvant or adjuvant therapy. They identified the depth of invasion as the most significant predictor of recurrence risk for squamous cell carcinoma, while tumor size was the primary predictor for adenocarcinoma. Furthermore, the presence of LVSI was associated with a higher risk of recurrence in adenocarcinoma. These findings can be used to develop a nomogram to assess an individual patient’s risk of recurrence. Based on these insights, the traditional risk factors for adjuvant therapy may need to be reconsidered.

The CERVANTES trial, which is a phase III randomized clinical trial, might resolve the questions regarding the role of adjuvant therapy in this group of patients. This study will measure the effectiveness of RT as an adjuvant treatment for patients with CC at intermediate risk of disease progression [38]. Patients with FIGO 2018 IB1-IIA squamous cell carcinoma or adenocarcinoma of the cervix, whose risk factors are relatively met, will be recruited for the trial. Radical hysterectomy and sentinel lymph node biopsy combined with pelvic lymphadenectomy will be the initial management, while patients will subsequently be randomized to either no further treatment or RT with or without chemotherapy or brachytherapy. The primary objective of the trial is the DFS rate at 3 years, while the secondary objectives are OS, survival free of pelvic disease, and health-related quality of life and side effects. Of course, there are still limitations to be mentioned. The trial could be affected by the nature of the data reported, namely the use of laparoscopy which, according to the LACC trial [39], is associated with worse oncological outcomes compared to open surgery. The consideration of the LACC trial raises the question of whether patients with intermediate-risk factors, undergoing minimally invasive surgery, warrant more aggressive adjuvant treatment compared to their open-surgery counterparts. Moreover, the data suggest that the current “intermediate-risk” category is overly heterogeneous. Different histological characteristics drive recurrence in adenocarcinoma and in squamous cell carcinoma moving towards a histology-specific risk stratification system. Furthermore, the fact that brachytherapy and chemotherapy are not uniformly administered as an adjuvant therapy may add further heterogeneity into the analysis. A further division of subgroups within intermediate-risk disease category, could safely guide precise de-escalation or escalation strategies. In any case, there is anticipation of the results of the CERVANTES trial, as it may potentially provide more definitive conclusions for how patients with intermediate-risk cervical cancer should be managed consequently.

## 5. Conclusions

In conclusion, achieving monotherapy in the treatment of CC still remains the pivotal goal in order to optimize patient outcomes with the relative minimization of overtreatment and avoidance of combination treatment morbidity. Regarding cases classified as IA1 and IA2, as well as low-risk IB1, IB2, and IIA1, adjuvant treatment is not typically necessary, and well-performed surgical treatment is associated with favorable outcomes. In contrast, CRT is absolutely indicated in high-risk cases with lymph node invasion, parametrial invasion, or a final tumor size over 4 cm. Intermediate-risk patients, namely those combining parameters such as a tumor size over 2 cm, positive LVSI, and greater depth invasion, represent a grey-zone category for which the largest prospective RCT indicated a benefit of adjuvant RT regarding PFS but not OS. However, increasing evidence stands in favor of comparable survival outcomes of an observation strategy in such cases, under the condition of an adequately performed surgery. Therefore, adjuvant RT or observation should be individualized in intermediate-risk cases. Advanced diagnostic modalities such as MRI or expert ultrasound, preoperative diagnostic conization, and potential surgical lymph node staging as the initial steps in treatment planning could facilitate the accurate identification of risk categories and the tailored implementation of monotherapy. Finally, to refine these treatment strategies, there is a high need for well-designed clinical trials incorporating standardized imaging techniques, histopathological evaluation, and updated RT protocols. Such trials may be able to balance the oncological benefit of adjuvant treatment with quality-of-life considerations, especially in intermediate-risk patients. Ultimately, these recommendations must be interpreted within the context of regional realities, as disparities in access to HPV vaccination, screening, and radiotherapy resources can significantly affect both adjuvant decision-making and the external validity of current evidence.

## Figures and Tables

**Table 1:** Risk stratification criteria across guidelines and mapping to Sedlis criteria.

Risk Category	Historical Sedlis (GOG-92, 1999)	ESGO 2018	ESGO 2023	NCCN 2025	Suggested Type of RH
Low Risk	Does not meet intermediate-/high-risk criteria	• Tumor < 2 cm • LVSI (−) • Stromal invasion: Inner 1/3	• Tumor < 2 cm • LVSI (−) • Stromal invasion: Superficial/minimal	• Tumor ≤ 2 cm • LVSI (−) • Stromal invasion: Superficial	A/B1
Intermediate Risk (Sedlis-Eligible)	Specific combinations required: • LVSI(+) + deep 1/3 invasion + any size • LVSI(+) + middle 1/3 invasion + tumor ≥ 2 cm • LVSI(+) + superficial 1/3 invasion + tumor ≥ 5 cm • LVSI(−) + middle or deep 1/3 invasion + tumor ≥ 4 cm	•Tumor ≥ 2 cm + LVSI(−) + any depth OR •Tumor < 2 cm + LVSI(+) + any depth	Any combination of: • LVSI(+) • Tumor ≥ 2 cm • Middle/deep 1/3 stromal invasion	Combination of risk factors: • LVSI(+) • Tumor > 4 cm • Deep stromal invasion	B2/C1
High Risk	• PLN metastases • Parametrial involvement • Positive margins	• Tumor ≥ 2 cm + LVSI(+) + any depth	• PLN metastases • Parametrial involvement • Positive margins	• PLN metastases • Parametrial involvement • Positive margins • Tumor ≥ 4 cm	C1/C2

LVSI, lymphovascular space invasion; PLN, pelvic lymph nodes; RH, radical hysterectomy; 2017 Updated Querleu–Morrow Classification of Radical Hysterectomy: Type A (Extrafascial/Simple Hysterectomy): minimal resection with paracervix removed halfway between cervix and ureter and minimal excision of ventral and dorsal parametrium; Type B1 (Modified Radical Hysterectomy): paracervix resected at the ureter level with ureter mobilized from cervix and lateral parametrium, partial excision of vesicouterine ligament ventrally, and partial resection of rectouterine–rectovaginal ligament and uterosacral fold dorsally; Type B2 (Modified Radical Hysterectomy with Lymphadenectomy): identical to B1 plus paracervical lymphadenectomy without resection of vascular or nerve structures; Type C1 (Radical Hysterectomy—Nerve-Sparing): paracervix resected at iliac vessels transversally with caudal part preserved, vesicouterine ligament excised at bladder with bladder nerves dissected and spared, and dorsal parametrium resected at rectum with hypogastric nerve spared; Type C2 (Radical Hysterectomy—Complete): complete paracervical resection at medial aspect of iliac vessels including caudal part, ventral parametrium resected at bladder with bladder nerves sacrificed, and dorsal parametrium resected at sacrum with hypogastric nerve sacrificed.

**Table 2:** High-risk cervical cancer: landmark trials –survival differences and grade ≥ 3 toxicity.

Study (Year)	Comparison	Follow-Up	PFS/DFS	OS	Grade ≥ 3 Acute Toxicity
Peters et al., 2000 [6]	CRT vs. RT alone (post-RH)	4 years	80% vs. 63% (HR = 2.01, *p* = 0.003)	81% vs. 71% (HR = 1.96, *p* = 0.007)	CRT 21% vs. RT 2.5% (*p* < 0.0001)
Kim et al., 2021 [10]	CRT + ACT vs. CRT alone (post-RH)	3 years (DFS) 5 years (OS)	80.7% vs. 85.0% (*p* = 0.539)	88.1% vs. 94.8% (*p* = 0.121)	-No difference in gastrointestinal toxicities except diarrhea (55.7% vs. 34.3%; *p* = 0.005) -Anemia and neutropenia more common in the study group
Weng et al., 2023 [12]	ACT vs. CRT (post-RH)	3 years (DFS) 5 years (OS)	91.9%vs 91.9%, HR = 0.854; (95% CI 0.415–1.757; *p* = 0.667)	90.6% vs. 90.0% Adjusted HR = 0.673; (95% CI 0.277–1.640, *p* = 0.384)	Grade 3–4 myelotoxicity slightly more frequent among patients in the 6-cycle chemotherapy group (*p* < 0.001)
Ma et al., 2023 [11] (meta-analysis)	ACT + CRT vs. CRT alone	5 years	HR = 0.81, 95%, CI: 0.67–0.96, *p* = 0.02	HR = 0.69, 95%, CI: 0.51–0.93, *p* = 0.01	ACT induced a greater rate of hematologic toxicities (*p* < 0.05)

RH, radical hysterectomy; CRT, chemoradiation; ACT, adjuvant chemotherapy.

**Table 3:** Eligible criteria of GOG92 trial [5].

Capillary Lymphatic Space Tumor Involvement	Stromal Invasion	Tumor Size
Positive	Deep 1/3	Any
Positive	Middle 1/3	≥2 cm
Positive	Superficial 1/3	≥5 cm
Negative	Deep or middle 1/3	≥4 cm

**Table 4:** Summary of adjuvant therapy studies in intermediate-risk cervical cancer patients.

Study	Design	Number of Patients	Criteria	Adjuvant Modality	Primary Endpoint	Effect on Primary Endpoints				Toxicity
Sedlis et al., 1999 [5]	RCT	277	-LVSI (+), deep 1/3 invasion, any size -LVSI (+), middle 1/3 invasion, size ≥ 2 cm -LVSI (+), superficial 1/3 invasion, size ≥ 5 cm; -LVSI (−), middle/deep 1/3 invasion, size ≥ 4 cm	Pelvic RT vs. Observation	Recurrence rate	Decreased recurrence rate with RT (15.3% vs. 27.9%, *p* = 0.008)		No significant differences in OS (*p* = 0.074)		Grade 3–4 toxicity: RT 7% vs. Observation 1.5%
Zhang et al., 2022 [13]	Meta-analysis	5052 (16 studies)	Early-stage cervical cancer after radical hysterectomy	Adjuvant CT vs. RT ± CCRT	DFS and OS	Adjuvant CT improved DFS (HR 0.77, 95% CI 0.62–0.92, *p* < 0.001; i2 = 0.0%)		Adjuvant CT improved OS (HR 0.69, 95%CI 0.54–0.85, *p* < 0.001; i2 = 0.0%)		Not reported
Lahousen et al., 1999 [14]	RCT	76	High-risk stage IB-IIB patients treated with radical hysterectomy with pelvic lymph node metastases ± vascular invasion	CT vs. Pelvic RT vs. Observation	DFS and OS	No significant differences in DFS		No significant differences in OS		Not reported
Tozzi et al., 2024 [15]	Prospective cohort	74	Nerve-sparing radical hysterectomy for FIGO IB3 patients	Observation only after nerve-sparing laparoscopic radical hysterectomy	DFS and OS	5-year DFS: 89.7%		5-year OS: 93.1%		Complication rate: 23.5% (all low grade)
Rotman et al., 2006 [16]	Phase III RCT	277	-Stage IB, -lymph nodes (-) -combination of deep stromal invasion (>1/3), LVSI (+), tumor size ≥ 4 cm	Pelvic RT vs. Observation	12-year OS and 12-year RFS	Improved RFS with RT: (HR = 0.58; *p* = 0.009)		No statistically different in OS (HR 0.85, *p* = 0.41)		Grade 3–4 toxicity: RT 8.3% vs. Observation 2.1% (*p* = 0.083)
Cibula et al., 2023 [17]	Retrospective subanalysis	692	N0 and combination of intermediate-risk factors (LVSI, tumor ≥ 2 cm, deep stromal invasion)	RT/CCRT vs. Observation	5-year DFS and OS	DFS (83.2% vs. 80.3%, *p* = 0.365)		OS (88.7% vs. 89.0%, *p* = 0.281)		Not reported
van der Velden et al., 2019 [18]	Retrospective cohort	161	Intermediate-risk, type C2 hysterectomy, no adjuvant therapy	Observation only	-5-year RFS -5-year OS -locoregional recurrence	5-year RFS: 86.6%	5-year OS: 90%		locoregional recurrence: 2.5%	Not reported
Cao et al., 2021 [19]	Retrospective cohort	861	Intermediate-risk factors (according to Sedlis criteria)	RT vs. CCRT vs. Observation	-5-year RFS -5-year DFS	No significant difference in RFS 87.1% vs. 84.2% vs. 89.6% (*p* = 0.27)		No significant difference in DFS 92.3% vs. 87.7% vs. 91.4% (*p* = 0.20)		Not reported
Nasioudis et al., 2021 [20]	Retrospective cohort	765	Stage IB with intermediate risk (FIGO 2018 IB1 with LVSI or IB2)	RT vs. Observation	4-year OS	No significant difference 88.4% vs. 87.1% (*p* = 0.44)				Not reported
Gómez-Hidalgo et al., 2022 [21]	Meta-analysis	1396 (8 studies)	Early-stage cervical cancer after radical surgery	RT vs. Observation	Recurrence and mortality risk	No significant difference in risk of recurrence (RR = 1, z = 1.29, *p* = 0.197)		No significant difference in mortality risk (RR = 1, z = 0.90, *p* = 0.366)		No significant differences in grade 3 and 4 adverse events
Rogers et al., 2012 [22]	Cochrane review	397 (2 studies)	Early cervical cancer after surgery	RT or CRT vs. Observation	5-year OS and PFS	No statistically significant difference in OS (HR 0.7; 95% CI 0.5 to 1.1)		Significantly lower risk of disease progression in RT (HR 0.6; 95% CI 0.4 to 0.9)		Higher risk for serious adverse events for RT compared to observation (not statistically significant)
Guo et al., 2022 [23]	Meta-analysis	3785 (14 studies)	Intermediate-risk factors (tumor > 2 cm, deep stromal invasion, LVSI)	CRT vs. RT alone	RFS, OS, toxicity or complications	Improved RFS rate with CRT: OR = 2.17, 95% CI [1.53, 3.07], *p* < 0.0001; i2 = 21%		No significantly improved OS rate with CRT: OR = 1.48 95% CI [0.97, 2.27], *p* = 0.07, i2 = 38%		Increased grade 3 or 4 hematological toxicity with adjuvant CRT (OR 7.73 95%, CI [3.40, 17.59], *p* < 0.0001; i2 = 62%)

LVSI, lymphovascular space invasion; RT, radiotherapy; CCRT, radiotherapy with concurrent chemotherapy; OS, overall survival; DFS, disease-free survival; PFS, progression-free survival; CRT, chemoradiation; RFS, recurrence-free survival.
